# The Intracellular Signaling Molecule Darpp-32 Is a Marker for Principal Neurons in the Cerebellum and Cerebellum-Like Circuits of Zebrafish

**DOI:** 10.3389/fnana.2016.00081

**Published:** 2016-08-04

**Authors:** Lena Robra, Vatsala Thirumalai

**Affiliations:** National Centre for Biological SciencesBangalore, India

**Keywords:** zebrafish, dopamine, cerebellum, Purkinje neurons, neuromodulation, optic tectum

## Abstract

The dopamine and cAMP regulated phosphoprotein of apparent molecular weight 32 kDa (Darpp-32) is an inhibitory subunit of protein phosphatase-1 (PP-1). Darpp-32 activity is regulated by multiple ligand-activated G-protein coupled receptors (GPCRs). This protein is coded for by the protein phosphatase-1 regulatory subunit 1b (*ppp1r1b*) gene. Here, we provide experimental evidence for the presence of multiple isoforms of *ppp1r1b* in zebrafish. We show that these isoforms are differentially expressed during development with the full-length isoform being maternally deposited. Next, with a custom polyclonal antibody generated against the full-length protein, we show that in the adult, Darpp-32 is strongly expressed in principal neurons of the cerebellum and cerebellum-like circuits. These include Purkinje neurons in the cerebellum, Type-I neurons in the optic tectum, and crest cells in the medial octavolateralis nucleus (MON). We confirmed the identity of these neurons through their colocalization with Parvalbumin 7 immunoreactivity. Darpp-32 is seen in the somata and dendrites of these neurons with faint staining in the axons. In all of these regions, Darpp-32-immunoreactive cells were in close proximity to tyrosine hydroxylase (TH) immunoreactive puncta indicating the presence of direct catecholaminergic input to these neurons. Darpp-32 immunoreactivity was seen in Purkinje neurons as early as 3 days post-fertilization (dpf) when Purkinje neurons are first specified. In sum, we show that Darpp-32, a signaling integrator, is a specific marker of principal neurons in the cerebellum and cerebellum-like circuits in zebrafish.

## Introduction

Neuromodulators act via G-protein coupled receptors (GPCRs) to alter cellular signaling states and firing properties. A key molecule involved in the integration of signaling via GPCRs is the dopamine and cAMP regulated neuronal phosphoprotein of 32 kDa (Darpp-32; Greengard et al., 1999; Svenningsson et al., 2004; Yger and Girault, 2011). Dopamine acting via D1 and D2 receptors and several other neuromodulators acting via their cognate receptors alter the phosphorylation status of Darpp-32 through cAMP and protein kinase A (PKA). When phosphorylated at threonine 34, Darpp-32 is a potent inhibitor of protein phosphatase-1 (PP-1) (Hemmings et al., 1984). As several GPCRs converge onto Darpp-32, this molecule acts as an intracellular signal integrator.

Darpp-32 was originally identified from rodent striatal extracts as a phosphoprotein regulated by dopamine (Walaas et al., 1983a,b). It is especially enriched in the striatal medium spiny neurons (MSNs; Ouimet et al., 1984), which are targets of dopaminergic neuromodulation from the substantia nigra. In mammals, Darpp-32 is predominantly expressed in the cell bodies, the dendrites and the terminals of dopaminoceptive neurons but not dopaminergic cells (Ouimet et al., 1984). In subsequent studies, it has become clear that Darpp-32 is an important signaling molecule not only for dopaminergic neuromodulation but also for several other modulatory systems (Svenningsson et al., 2004). To understand neuromodulatory control of target circuits, it therefore becomes imperative to map Darpp-32 expression in these circuits.

Zebrafish are an excellent model system to study the development, functioning and neuromodulation of neural circuits because of the availability of several tools like molecular genetics, imaging and electrophysiology. Being a vertebrate, the overall organization, development and neuromodulatory pathways are conserved in zebrafish compared to mammals. Catecholamines (dopamine, adrenaline and noradrenaline) are a major source of neuromodulation within the central nervous system (CNS). The catecholaminergic system in teleosts has been evaluated in numerous studies mostly through expression of the rate-limiting enzyme of catecholamine synthesis, tyrosine hydroxylase (*th1*; Rink and Wullimann, 2002; McLean and Fetcho, 2004a,b; Tay et al., 2011). Recently additional catecholaminergic regions were characterized, based on the expression of another copy of th, *th2* (Candy and Collet, 2005; Filippi et al., 2010). However identification of regions and cell types that are the target of aminergic innervation and modulation remains incomplete. Evaluating the expression pattern of Darpp-32 in the zebrafish will help in the identification of dopaminoceptive and adrenoceptive neuroanatomical structures.

The cerebellum is one of the most primitive structures in the vertebrate brain and is required for motor adaptation and motor learning. In addition, most vertebrates also have cerebellum-like circuits that receive parallel fiber inputs in their molecular layers and peripheral inputs from deeper layers converging onto principal neurons (Bell, 2002; Bell et al., 2008). In the cerebellum, these principal neurons are the Purkinje neurons. We have recently shown that many of the cellular properties of cerebellar Purkinje neurons are conserved from zebrafish to mammals. Furthermore, Purkinje neurons in zebrafish represent an efference copy of the motor commands in their activity (Sengupta and Thirumalai, 2015), consistent with the cerebellum playing important roles in motor co-ordination and learning. In addition to the cerebellum, cerebellum-like circuits are present in the medial octavolateralis nucleus (MON) and the optic tectum of zebrafish (Bae et al., 2009). All of these circuits receive rich innervation from catecholaminergic neurons (Kaslin and Panula, 2001; McLean and Fetcho, 2004a,b; Tay et al., 2011), suggesting a significant role for Darpp-32 in these circuits. In this study, we show that Darpp-32 is strongly expressed in all principal neurons of the cerebellum and cerebellum-like circuits underlining the significance of Darpp-32 in modulating function in these analogous circuits.

## Materials and Methods

### Maintenance of Zebrafish

Adult zebrafish (*Danio rerio*, Indian wild type) were obtained from a commercial supplier and housed in a recirculating water system (Tecniplast, Italy), maintaining a 14 h light/10 h dark cycle. Adult fish were used for the generation of embryos. All procedures were approved by the Institutional Animal Ethics Committee, National Centre for Biological Sciences. In total 10 adult zebrafish brain were used for sagittal and coronal sections and between 5 and 15 larva have been used per stage for whole mount immunofluorescence staining. Twelve larvae per stage have been used for *in situ* hybridization (three larvae for sense probe and three larvae for antisense probe per experiment).

### RNA Isolation

RNA was isolated from zebrafish larval and adult zebrafish brain using the RNeasy Mini kit (Qiagen, New Delhi). One adult zebrafish (Indian wild type) was euthanized by submersion in ice-cold 0.1% w/v ethyl 3-amino-benzoate methane sultanate (MS222, Sigma Aldrich, Electronics city, Bengaluru). The brain was dissected in ice-cold normal Ringer’s solution (116 mM NaCl, 2.9 mM KCl, 1.8 mM CaCl2, 5.0 mM, HEPES, pH: 7.4) using fine forceps (Fine Science Tools, F.S.T., North Vancouver, BC, USA). The cerebellum was separated from the rest of the brain using a micro scalpel (F.S.T.). The part of the brain anterior to the cerebellum and the cerebellum were transferred into separate 1.5 ml reaction tubes and snap frozen by submersion in liquid nitrogen. The tissue was powdered using a single use plastic pestle (Sigma Aldrich) and the RNA was extracted according to the manufacturers’ manual. RNA from embryos and larvae of the respective stages was isolated using the same kit, according to the instructions in the manual. For stages up to 4 days post-fertilization (dpf), 10 embryos were pooled and used for RNA extraction. For 4 dpf and 6 dpf larvae only the heads of 10 larvae were pooled for RNA extraction. RNA concentration was measured at a Nanodrop spectrophotometer (Thermo Scientific). RNA quality was assessed by running a 1 μg aliquot of RNA on a 2% w/v agarose gel in 1× Tris-acetate-EDTA (TAE)-buffer at 4.75 V/cm for 45 min. The RNA was stained with 0.5× GelRed (Biotium, Hayward, CA, USA) and visualized using UV light and a GelDoc system (Labindia Instruments, Malleswaram, Bengaluru).

### cDNA Preparation

RNA was retro-transcribed into cDNA using SMARTScribe Reverse transcriptase (DSS Takara Bio India Pvt. Ltd., New Delhi) according to the manufacturer’s instructions. Up to 1 μg of RNA was used per 20 μl reaction. After the retro-transcription, 20 μl of Tricine-EDTA buffer was added to the reaction and the cDNA was stored at −20°C until further usage.

### Amplification of *ppp1r1b* mRNA

The annotated sequences for the phosphoprotein protein phosphatase1 regulatory subunit 1b (*ppp1r1b*) gene in the latest release of the zebrafish genome (GRCz10) stored in the Ensembl database^1^ were used for designing gene specific oligonucleotide primers. The primers were designed to bind in the 5′ and 3′ untranslated region (UTR) of the transcripts. *ppp1r1b* transcript 1 and 2 (*ppp1r1b*-*001* and *ppp1r1b*-*002*) were amplified from cDNA using the following oligonucleotides: Forward: 5′ GCGTGTATGTACTGTTCAGACAGGCA 3′ Reverse: 5′ AAGGAACAGCATCGT AGCACTCCATA 3′. The transcript *ppp1r1b*-*003* was amplified using the following oligonucleotides: Forward: 5′AAGTGGGTCGGAGAGCTGTTCTGTA 3′ and Reverse: 5′ AAGGAACAGCATCGTAGCACTCCATA 3′. The transcripts were amplified using Herculase DNA Polymerase (Agilent, Mahadevapura Post, Bengaluru) with the following cycling conditions: 95°C for 1 min, 40 cycles of (95°C for 20 s, 68°C for 1 min) and a final extension at 68°C for 5 min. The product was run on a 2% w/v agarose gel containing 0.5× GelRed in 1× TAE buffer, then gel eluted using a gel extraction kit (Qiagen) and cloned into the pCR^®^-Blunt vector (Invitrogen, Whitefield, Bengaluru). Putative positive clones were identified through colony Polymerase chain reaction (PCR) with oligonucleotides complementary to the M13 promoter (oligonucleotides provided in the kit). In brief, single colonies were transferred onto a grid plate. The same colonies were used for a PCR, using OneTaq Hotstart polymerase (NEB, Gurgaon) with the following cycling conditions: 94°C for 5 min, 35 cycles of (94°C for 30 s, 52°C for 30 s, 68°C for 60 s) and a final extension at 68°C for 5 min. Putative positive colonies were identified on a 1.5% w/v agarose gel and grown over night at 37°C, shaking at 200 rpm in 5 ml Luria Bertani (LB) medium with Kanamycin (50 mg/ml) as selection agent. The plasmids were isolated using the Qiagen Miniprep kit (Qiagen) and sequenced at the in-house sequencing facility using oligonucleotides complementary to the M13 promoter. The same PCR conditions as for the cloning were used for establishing the developmental profile of *ppp1r1b* expression. One microliter of cDNA was used per 20 μl reaction. A 620 bp fragment of *beta-actin 1/2* (*actb1/2*) was amplified as control for cDNA quality and input quantity. The following oligonucleotides were used for amplification of beta-actin 1/2: Forward: 5′ ATCGACCACGGTATTGTCACCAACTG 3′ and Reverse: 5′GTGGTCTCGTGGATACCGCAAGATT 3′. The cycling conditions were as follows: 95°C for 1 min, 25 cycles of (95°C for 30 s, 55°C for 30 s, 68°C for 1 min) and a final extension for 5 min at 95°C.

### Identification of Transcription Start Sites using 5′ Rapid Amplification of cDNA Ends (RACE)

The SMART RACE cDNA amplification kit (Clontech/DSS Takara Bio India Pvt. Ltd., New Delhi) was used to generate first-strand cDNA complementary to mRNA starting from the intact 5′ end. This 5′ RACE cDNA was used for subsequent amplification with internal reverse oligonucleotides that are specific to the *ppp1r1b* gene. The following reverse oligonucleotides were used: 5′RACE 1: 5′ CGATTCAGTTGTGCAGGATGAGCAGA 3′ and 5′RACE 3: 5′ GGAGGTAATGTCGTCCTCAGGAGATG 3′. The PCR reaction was loaded onto a 2% w/v agarose gel containing 0.5× GelRed in 1× TAE buffer. The product was gel eluted using a gel extraction kit (Qiagen) and cloned into the pCR^®^-Blunt vector (Invitrogen, Whitefield, Bengaluru). Putative positive clones were identified and grown as described above. The plasmids were isolated using the Qiagen Miniprep kit (Qiagen) and sequenced at the in-house sequencing facility using oligonucleotides complementary to the M13 promoter. The transcription start sites (TSSs) were identified by alignment with the genomic region of *ppp1r1b* using Benchling^2^.

### Whole Mount RNA *In Situ* Hybridization

The *in situ* probe was generated using the *ppp1r1b-001* cloned into the pCRII vector. This vector allows transcription of the antisense probe, using the T7 promoter after linearization with the restriction enzyme Hind III and transcription of the sense probe, using the SP6 promoter after linearization with the restriction enzyme XhoI. For each probe generation 5 μg of plasmid were linearized and gel purified. RNA was transcribed according to the manufacturers manual using the mMessage machine kit (Ambion) and purified using a RNA clean up kit (Qiagen). Probe integrity and size was checked by running a 5 μl aliquot of purified RNA on a 1.5% TAE gel, containing 0.5× GelRed.

Larvae were staged based on their external morphological features (Kimmel et al., 1995) and subsequently fixed in 4% Paraformaldehyde (PFA) in phosphate buffered saline (PBS) at 4°C over night. Embryos older than 16 hours post fertilization (hpf) were dechorionated prior to fixation. For stages older than 24 hpf, 1× Phenylthiourea (PTU) was added to the medium to prevent pigmentation. PTU containing medium was replaced every 2 days. The next day embryos were washed 3 times 10 min with PBS. PBS was replaced with Hybridization solution and kept for 3–5 h or overnight at 65°C in the water bath. Processed embryos were stored at −20°C until all stages of larva were ready for subsequent procedure. Embryos older than 16 hpf were subjected to proteinase K treatment prior to the pre hybridization step. For that a freshly prepared solution of 10 μg/ml proteinase K in PBS with Tween 20 (PBST) were added to the larvae and incubated at room temperature for the following time periods: 24 hpf for 5 min, 30 hpf for 7 min, 48 hpf for 10 min, 72 hpf for 15 min, 96 hpf for 17 min, 5 dpf for 20 min. The larva were then rinsed 3 times with PBST and postfixed with 4% PFA for 20 min at room temperature. For the hybridization step the probe was prepared by adding 1 μl probe per 100 μl hybridization solution and heating this mixture for 5 min to 80°C and cooling it for 2 min on ice. Larvae were incubated over night at 65°C with this probe solution, sibling larvae were processed with sense and antisense probe in parallel. The larvae were then washed with a decreasing gradient of hybridization solution and increasing gradient of 2× SSCT (0.3 M NaCl, 0.03 M sodium citrate, 0.1% Triton X 100) for 15 min each at 65°C, finishing with a last step of 2× 30 min wash with 0.2× SSCT at 65°C. The larvae were washed at room temperature with a decreasing gradient of 0.2× SSCT and increasing gradient of maleic buffer, each step lasted 5 min. The larvae were then blocked for 1 h at room temperature with 1× blocking reagent in 1× maleic buffer (0.1 M maleic acid, 0.15 M NaCl). The samples were then incubated with anti-DIG antibody (1:5000) in blocking solution over night at 4°C. To develop the stain, the samples were first washed 4× 20 min in 1× maleic buffer, then washed 3× 5 min with staining buffer (1M Tris HCl pH 9.5, 5 M NaCl, 1 MgCl_2_, 10% Tween 20) then incubated with NBT and BCIP in staining buffer at room temperature in the dark. Development of the stain was checked on the microscope and reached its maximum stain after 30 min. Embryos were washed thoroughly with PBS and transferred into 80% glycerol and left over night at 4°C for equilibration. Embryos were imaged under a stereomicroscope on a white background with an additional light source from the top.

### Multiple Sequence Alignment

For the multiple sequence alignment (MSA) of zebrafish Darpp-32 with human, rat and mouse DARPP-32 the respective protein sequences were downloaded from www.ensembl.org. The sequences were submitted to MUSCLE 3.8, a web tool provided by EMBL-EBI^3^ (Edgar, 2004). The output file was used to calculate the conservation score and generate the graphic representation in Jalview (Waterhouse et al., 2009).

### Antibody Generation and Verification

The full-length mRNA for *ppp1r1b* coding DNA sequence (cds) was cloned into the pGEX vector (GE Healthcare, UK), to code for a glutathione-S-transferase (GST)-Darpp-32 fusion protein. Custom polyclonal antibodies to zebrafish Darpp-32 were generated in a rabbit host by a commercial vendor (Bioneeds, Bangalore). The antibodies were affinity purified and subsequently tested for functionality and specificity. To test the specificity of the antibody the *ppp1r1b* transcripts 1, 2 and 3 were cloned into the pEGFP N1 vector (DSS Takara Bio India Pvt. Ltd., New Delhi, GenBank accession: #U55762), to generate an N-terminal fusion protein with enhanced green fluorescent fusion protein (EGFP). To generate the fusion protein, the *ppp1r1b* cds was amplified from the pCR BLUNT vector containing the *ppp1r1b* sequence using the following oligonucleotides: Forward: 5′ ATAGCTAGCATGGATCCTTCGAGTCCCTC 3′ and Reverse 5′ ATACTCGAGGTCCAGATTTCTGCTCTCCTC 3′. The forward oligonucleotide contains a restriction enzyme recognition site for NheI (NEB, Gurgaon) and the reverse oligonucleotide contains a restriction enzyme recognition site for XhoI (NEB, Gurgaon). These two sites were employed to clone the *ppp1r1b* coding sequence (cds) into the pEGFP N1 vector. One microgram of the plasmid was subsequently transfected into HEK 293T cells that were seeded on confocal dishes. The cells were washed with 0.1 M PBS and fixed with 4% PFA (Alfa Aesar, Johnson Matthey Chemicals, Chennai) for 15 min at room temperature, 24 h after transfection. The cells were subsequently permeabilized using 0.2% Triton X 100 (USB Corporation, Cleveland, OH, USA) in PBS and blocked for 30 min using Blocking Reagent (Sigma Aldrich). The cells were incubated over night at 4°C with anti-Darpp-32 antibody (1:1000) diluted in blocking solution. Then, the cells were rinsed 3 times with PBS and incubated for 1 h at room temperature in the dark with anti-rabbit IgG coupled with Alexa 568 (1:1000, Invitrogen, transcript *ppp1r1b-001* and *ppp1r1b-002*) or with anti-rabbit IgG coupled with Alexa 649 (1:1000, Invitrogen, transcript *ppp1r1b-003*). The cells were then rinsed and overlaid with PBS and imaged at an Olympus FV 1000 confocal microscope, using a 40× oil immersion objective (transcript *ppp1r1b-001* and *ppp1r1b-002*) or a 60x oil immersion objective (transcript *ppp1r1b-003*). As a control, HEK 293T cells were transfected with equal amounts of pEGFP-N1 only and processed in the same way as described above. To show that the staining is not due to unspecific binding of the secondary antibody, pEGFP-N1-*ppp1r1b* transfected HEK293T cells were stained with secondary antibody only.

To further validate the specificity of the antibody, immunoprecipitation from whole brain lysate was performed and the pulled down protein was analyzed using mass spectrometry and western blotting. For this, brains from two adult zebrafish were dissected out after euthanasia in ice-cold 0.1% w/v MS-222. The brains were transferred into 1.5 ml reaction tubes and immediately snap-frozen in liquid nitrogen. The brains were then submerged in RIPA-buffer (150 mM NaCl, 1% Triton-X 100, 0.5% Sodium deoxycholate, 0.1% sodium dodecyl sulfate (SDS), 50 mM Tris, pH 8) with 1× protease inhibitor cocktail (PIC, Sigma Aldrich) and mechanically homogenized using a motor-driven homogenizer. The homogenate was centrifuged for 20 min at 4°C at 12,000 rpm. The protein concentration in the supernatant was determined using Bradford test (Bradford, 1976). Approximately 1 mg of total protein was used for the following immunoprecipitation with the custom-made Darpp-32 antibody. For the immunoprecipitation, blocked and precleared sepharose A beads were incubated with the Darpp-32 antibody for 3 h at 4°C. The antibody-coated beads were subsequently incubated over night at 4°C with the zebrafish whole brain lysate. The beads were subsequently washed 2 times with PBS containing 1× PIC followed by two washes with PBS with 0.1% TritonX-100 and 1× PIC. To analyze the immunoprecipitation, the precipitate was separated using 15% SDS-polyacrylamide gel electrophoresis (SDS-PAGE). For the western blot analysis, proteins were transferred to a nitrocellulose membrane using a BioRad Wet blotting system (Bio-Rad, Bangalore, India). The membrane was subsequently blocked in 1× Blotto (Santa Cruz Biotechnology, Dallas, TX, USA) in PBS and incubated with the Darpp-32 antibody (1:50) in 1× Blotto over night at 4°C. The membrane was then incubated with anti-rabbit IgG conjugated to HRP (1:1000). Protein bands were visualized using HRP-substrate (Amersham) and the ImageQuant 4000 (GE Healthcare life sciences, Bangalore). For the mass spectrometric analysis the SDS-PAGE was stained with Coomassie. The stained protein was cut from the gel and given for further processing to the mass spectrometry facility. Mass spectrometry was performed after trypsin digestion of the protein. The results were analyzed using the software PEAKS 7.0 (Bioinformatics solutions Inc., Waterloo, ON, Canada). Peptide sequences obtained were aligned and the results tabulated with number of unique sequences obtained for each hit using the same software. The false discovery rate was set to 0.6%.

### Whole Mount Immunofluorescence Staining of the Larval Zebrafish Brain

Larval zebrafish were staged as described above. Larvae of the respective stages were collected in a 1.5 ml reaction tube and anesthetized with 0.01% MS222. The larvae were then placed one by one into freshly prepared 4% PFA in PBS and collected in a fresh tube with 4% PFA and 0.005% Tween 20. The larvae were fixed over night at 4°C on a rocking platform. The next day the larvae were washed several times with PBST (0.1 M phosphate buffered saline and 0.1% Triton X 100). For dissection of the larva brain, the larva was pinned onto a petri dish coated with Sylgard (Dow Corning, Midland, MI, USA) using pins made from 0.001″ Tungsten wire (California Fine Wire Company, Grover Beach, CA, USA). First the skin of the head was carefully peeled off, and then the eyes and jaws were removed. Brains of the same stage were collected in the wells of a 96-well plate and incubated with 5% v/v normal donkey serum (NDS, Jackson Immuno Research, West Grove, PA, USA) in PBST for 3 h at room temperature. The primary antibody was added in the respective concentration and the brains were incubated 48 h at 4°C on a rocking platform. The brains were washed several times with PBS and incubated with the respective secondary antibody over night at 4°C on a rocking platform. The brains were rinsed with PBS and mounted in ProLong Gold Antifade reagent (Invitrogen) between two coverslips. The coverslips were sealed with clear nail polish (Lakme, India) and subsequently imaged at the Olympus FV 1000 confocal microscope. Antibody dilutions: anti-Darpp-32, custom made, 1:1000; anti-TH 1:1000; anti-Pvalb7, MAB1572 (Millipore, Bengaluru), 1:2000; anti-rabbit IgG coupled with Dylight 488, Jackson Immuno Research 1:1000; anti-mouse IgG coupled with Dylight 649, 1:1000).

### Immunofluorescence Staining of Adult Zebrafish Brain

Adult zebrafish were deeply anesthetized in ice-cold 0.1% MS 222. The fish was kept submerged in ice-cold normal Ringer’s solution and decapitated. The brain was dissected out of the skull and immediately kept in 4% freshly prepared ice- cold PFA. The brain was fixed overnight at 4°C. The next morning the brain was washed several times with PBS and then subjected to a sucrose gradient of 5%, 10%, 15% and 20% sucrose in PBS, each incubation lasting 30 min, except the last step, which was overnight at 4°C. The next day the brain was transferred to a 1:1 mixture of Jung tissue freezing medium (Leica, Nussloch, Germany) with 20% sucrose in PBS and incubated for 1 h at room temperature. The brain was then frozen in Jung tissue freezing medium by submerging it in liquid nitrogen vapor or on dry ice and mounted for cryosectioning. The brain was either oriented for sagittal or coronal sections. The brain was sliced into 14–20 μm sections and the sections were directly collected onto gelatine (3%) coated specimen slides. The slides were either air-dried at room temperature over night or for 30 min at 37°C on a hotplate. The slides were rehydrated with PBST (PBS, 0.1% Triton X 100). They were then washed twice with PBS-DT (PBS, 1% BSA, 1% DMSO, 1% Triton X 100) followed by a 1 h incubation with 5% NDS in PBS-DT at room temperature. Immediately thereafter the slides were incubated with primary antibody solution (antibody in 5% NDS/PBST) over night at 4°C. Antibody dilutions were the same as for whole mount immunofluorescence. The next day the slides were washed 6 times with PBS-DT and subsequently incubated with the respective secondary antibody overnight at 4°C in 5% NDS/PBST). After that the slides were washed six times with PBS-DT, mounted in mounting medium (80% glycerol, 2% n-propyl gallate (NPG, Sigma Aldrich) in PBS), and sealed with a coverslip and nail polish. The slides were stored in the dark at 4°C until imaging.

### Image Acquisition and Processing

Images were acquired using the Olympus FV 1000 confocal microscope. Images were taken using either a 10× or 20× air objective or a 40× or 63× oil immersion objective. Images were processed offline using the open source image processing package Fiji^4^. Fiji was used to adjust the brightness and contrast, to crop the image to the region of interest and to collapse single optical slices into a *z*-project as indicated. Scale bars were added based on the metadata. Stitching of images was either done using the pairwise stitching plugin in Fiji (Preibisch et al., 2009).

## Results

### Gene Structure and mRNA Expression

**Figure 1:**
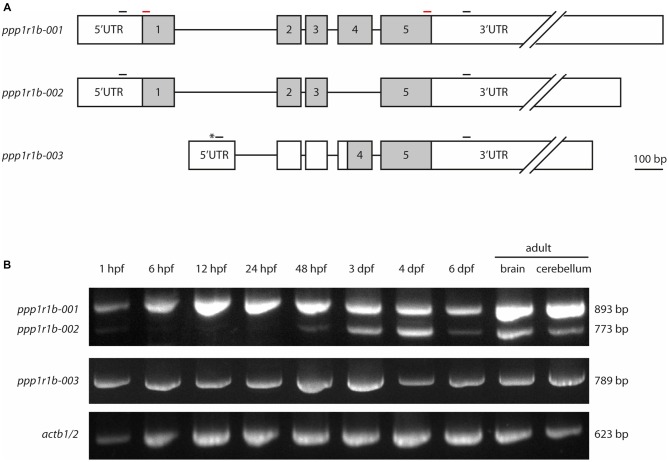
**Protein phosphatase1 regulatory subunit 1b (*ppp1r1b*) gene structure, isoforms and expression during development. (A)** Three isoforms of *ppp1r1b* were identified in zebrafish. Shaded areas designate the coding exons, non-shaded areas designate the untranslated regions (UTRs). Asterisk indicates an alternate transcription start site (TSS). Small black bars indicate positions of the primers used for RT-PCR. Red bars indicate positions of primers used to synthesize *in situ* hybridization probes. **(B)** RT-PCR of the different *ppp1r1b* isoforms during multiple stages of development. *actb1/2* was used as quality control for the cDNA synthesis. The exon lengths are drawn to scale, the intron lengths are representative of size but do not scale to the scale bar.

The gene encoding Darpp-32, *ppp1r1b*, belongs to the family of protein phosphatase 1 regulatory subunits (*ppp1r1*). The gene is annotated in the zebrafish genome in multiple databases (Ensembl, NCBI and ZFIN); however, there are no published studies giving experimental evidence for the transcription and expression of the *ppp1r1b* gene in zebrafish. There is also ambiguity in the different databases regarding the number of isoforms. The databases of the European Bioinformatics Institute and the Wellcome Trust Sanger Institute, Ensembl^5^ lists three transcripts under *ppp1r1b*, annotated as *ppp1r1b-001*, *ppp1r1b-00*2 *and ppp1r1b-003* on chromosome 19 (Figure 1A) while the National Center for Biotechnology Information (NCBI^6^, lists only two isoforms for *ppp1r1b* in the zebrafish genome: NM_001281923.1 and NM_001281924.1. Those two isoforms refer to *ppp1r1b-001* and *ppp1r1b-002* annotated in Ensembl respectively. The principal isoform, *ppp1r1b-001*, is 4516 base pairs (bp) long and encodes a 189 amino acid (aa) long protein. The second isoform, *ppp1r1b-002*, is 998 bp long and encodes a protein of 149aa in length. *ppp1r1b-002* has a shorter 3′ UTR compared to *ppp1r1b-001* and lacks exon 4. Both isoforms share the same 5′ UTR and TSS. The third isoform *ppp1r1b-003* has a unique exon 1 located in the intronic region between exon 1 and 2 of isoforms *ppp1r1b-001* and *ppp1r1b-002* and contains a unique TSS (Asterisk in Figure 1A). The remaining exons and introns are the same as in *ppp1r1b-001* and *ppp1r1b-002* except that the 3′ UTR is shorter than those of *ppp1r1b-001* and *ppp1r1b-002*. *ppp1r1b-001* encodes the full length protein while the protein encoded by *ppp1r1b-002* lacks exon 4 and is 40 aa shorter than the full length protein. Nevertheless, both isoforms share the same N-and C-terminus. The isoform *ppp1r1b-003* codes for a protein product of 94 aa. We could identify the TSS of all three isoforms using 5′RACE PCR, blunt end cloning of the PCR product and subsequent Sanger sequencing. Using Sanger sequencing, we identified multiple single nucleotide polymorphisms (SNPs) that repeatedly occurred across multiple samples but not all. The most consistent ones were synonymous substitutions (at amino acid residue 20 the codon CAA (Q) changed to CAG (Q), at amino acid residue 87 the codon CCC (P) changed to CCA (P), brackets indicate the aa encoded by the codon before the bracket. In addition, two non-synonymous substitutions were observed. One is a change from glutamic acid (E) to glycine (G) at amino acid residue position 50 and the other a change from histidine (H) to arginine (R) at amino acid residue position 65. Both of these are relatively drastic changes with respect to their physico-chemical properties, hence it is expected that they will affect the mature protein. However, these changes are in the C-terminal part of the protein that is not primarily involved in the interaction with PP1. A common observation is a variation in the length of glutamic acid (E) stretch in the C-terminus of the protein. UniProt^7^ lists the following entries for zebrafish Darpp-32: E7F7T1 for *ppp1r1b-001* and X1WG03 for *ppp1r1b-003* respectively.

To investigate the role of Darpp-32 during development, and to see whether the different isoforms might play different roles during development, we designed primers to establish a developmental expression profile for all three isoforms. PCR amplification from cDNA yielded three distinct products of 893 bp, 789 bp and 773 bp in length. Sanger sequencing and alignment of the sequences with the annotated *ppp1r1b* transcripts of these products identified them as *ppp1r1b-001*, *ppp1r1b-002* and *ppp1r1b-003*. Amplification from cDNA generated from different time points of development shows that *ppp1r1b-001* and *ppp1r1b-003* are maternally deposited and expressed throughout advancing stages of development (Figure 1B). *ppp1r1b-002* is very faintly visible at 1 hpf and is thereafter not detectable till 48 hpf, at which point, it starts to be endogenously expressed (Figure 1B). The ratio of *ppp1r1b-001* to *ppp1r1b-002* expression is biased towards *ppp1r1b-001* throughout development, with the bias most prominent in the adult brain/cerebellum. The expression of *ppp1r1b-003* is stable throughout development (Figure 1B). Note, that this study is only looking at the qualitative expression profile of *ppp1r1b*. Therefore, it is unlikely that the weaker intensities of *ppp1r1b-001* and *ppp1r1b-002* at 6 dpf (Figure 1B) reflect an actual change in gene expression, but are more likely a result of variability of efficiency of the PCR.

### Whole Mount RNA *In Situ* Hybridization

**Figure 2:**
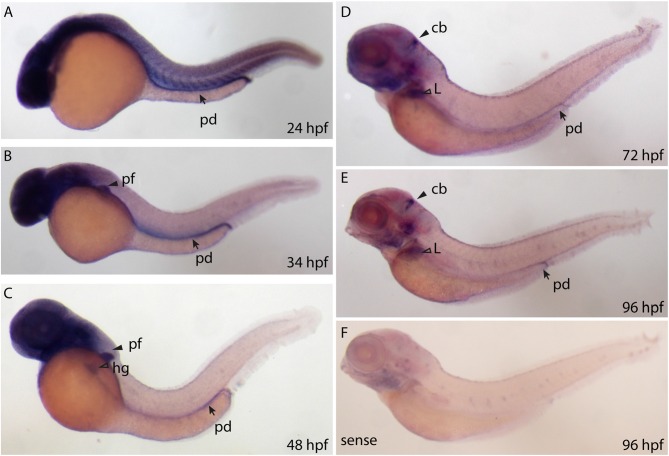
**Whole mount RNA *in situ* hybridization using probes against the *ppp1r1b-001* isoform at different stages of development. (A)** 24 hours post fertilization (hpf) embryo showing ubiquitous staining including in the pronephric duct (pd). **(B)** More restricted staining in the central nervous sytem (CNS) and the pectoral fin buds (pf). **(C)** At 48 hpf, faint staining is also seen in the hatching gland (hg). **(D)** At 72 hpf, strong staining is seen in the cerebellum (cb), liver (L) and the pronephric duct (pd). **(E)** A similar staining pattern is seen at 96 hpf **(F)** 96 hpf larva stained with the sense probe.

We designed a digoxigenin (DIG)—labeled mRNA probe that spans the full length *ppp1r1b-001* sequence to establish a spatio temporal expression profile of *ppp1r1b* mRNA in the developing zebrafish larva. *ppp1r1b* mRNA was detected uniformly at shield stage (6 hpf, data not shown). At 24 hpf *ppp1r1b* mRNA was detected in the CNS, in the myotomes and in the developing pronephric duct (Figure 2A). At 34 hpf, *ppp1r1b* mRNA is no longer expressed in the myotomes. Expression in the pronephric duct and in the CNS continues. Additional expression of *ppp1r1b* mRNA is visible in the developing pectoral fin (pf, Figure 2B). At 48 hpf the CNS, pectoral fin and hatching gland (hg) continue to express *ppp1r1b* mRNA (Figure 2C). From 72 hpf onwards the expression of *ppp1r1b* mRNA in the CNS concentrates in the cerebellum (cb, Figure 2D). Additionally expression of *ppp1r1b* mRNA is visible in the developing liver and in the pronephric duct. This expression pattern continues at 96 hpf (Figure 2E). The sense probe shows weak labeling in the head region and in the neuromasts of the lateral line (Figure 2F). The same pattern is visible with the antisense probe. We therefore conclude that this pattern is unspecific background staining.

### Multiple Sequence Alignment of Darpp-32 Protein Sequences

The subdomains required for Darpp-32 mediated inhibition of the catalytic subunit of protein phosphatase 1 (PP1c) have been previously identified in human, rat and mouse (Huang et al., 1999). This study identified the peptide sequences important for PP1c-DARPP-32 interaction as RRRRPTPAMLF (subdomain 1) and KKIQF (subdomain 2), with subdomain 2 being more N-terminal than subdomain 1. Subdomain 1 contains the phosphorylation site important for PP1c inhibition. It binds to the active site of PP1c, thereby preventing substrates of PP1c to enter the active site. Subdomain 2 binds in a hydrophobic groove of PP1c remote from the active site (Huang et al., 1999). Both subdomains are required for maintaining PP1c inhibition.

**Figure 3:**
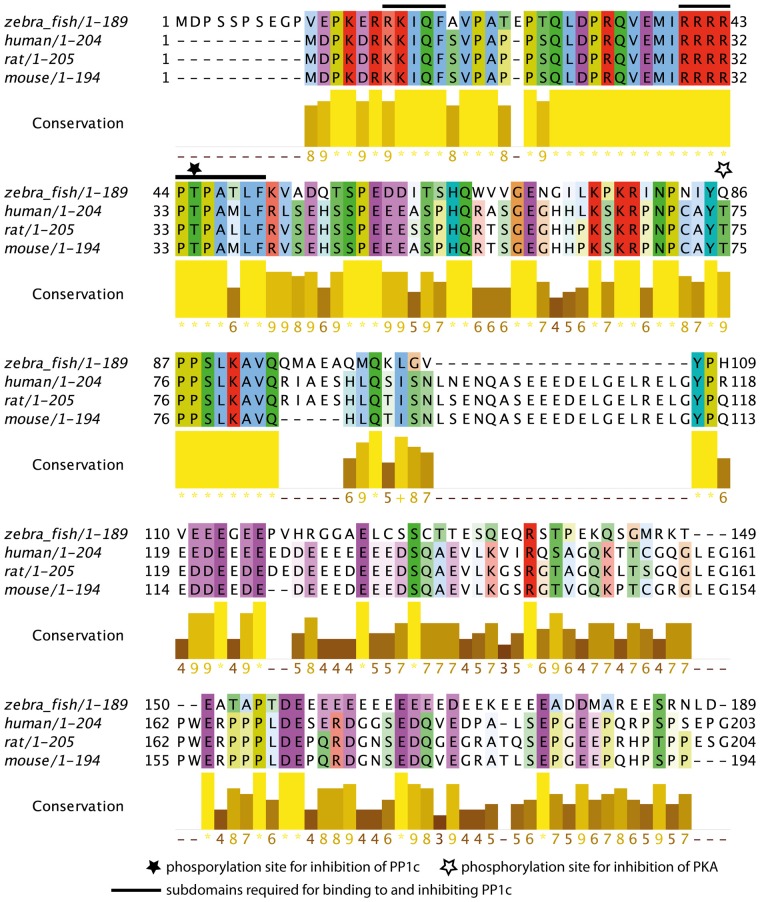
**Multiple sequence alignment (MSA) of zebrafish Darpp-32 with human, rat and mouse DARPP-32.** The color-coding is according to Clustalx; in this amino acid residues are shaded based on their physicochemical properties. The intensity of color indicates degree of conservation across the alignment in that position. The conservation score is calculated based on the physicochemical properties as well, asterisks represents 100% conservation and hyphen represents 0% conservation (Livingstone and Barton, 1993). The N-terminus (amino acid residues 11–94) of the protein is highly conserved across those 4 species. The phosphorylation site required for catalytic subunit of protein phosphatase 1 (PP1c) inhibition is marked with a black star (T-45, numbering refers to zebrafish sequence). The subdomains required for binding to and inhibiting PP1c as reported by Huang et al. (1999) are marked with a black bar (amino acid residues 17–21 and 40–50, numbering refers to the zebrafish sequence). A second phosphorylation site that leads to inhibition of protein kinase A (PKA) in human, mouse and rat is marked by a white star. This site is not present in zebrafish, however, the immediate surrounding of this residue is highly conserved. The MSA was done using MUSCLE 3.8 (Edgar, 2004) and the calculation of the conservation score and the graphic representation was done in Jalview (Waterhouse et al., 2009).

To see whether these subdomains are also present in the zebrafish protein we did a MSA of the zebrafish Darpp-32 amino acid sequence with human, rat and mouse Darpp-32 amino acid sequences (Figure 3). We find the phosphorylation site required for inhibition of PP1c and the surrounding motif highly conserved (T-34 in human, rat and mouse, T-45 in zebrafish, black star and black bars in Figure 3). Zebrafish Darpp-32 has additional 10 N-terminal amino acids, as compared to human, rat and mouse Darpp-32. This causes a shift of the threonine residue required for activity towards the C-terminus. The function of this N-terminal addition is not known and could not be mapped to any known domain. Overall, the MSA shows that the subdomains required for PP1 interaction are present but slightly altered in zebra fish (Figure 3). The zebra fish subdomain 1 is RRRRPTPA**T**LF and the subdomain 2 is **R**KIQF (altered amino acids are shown in bold letters, see also black bars in Figure 3). The first substitution is at position 17 and replaces a lysine residue with an arginine residue. Though Huang et al., 1999 define this lysine to be important for inhibitory function, they also state that a degenerated motif BB(V/I)XF will be sufficient for binding of the subdomain 2 (Huang et al., 1999). Since arginine has similar physicochemical properties to lysine, it is likely that the subdomain 2 of the zebrafish Darpp-32 will bind in a similar matter as the motif reported for human, rat and mouse. The second change is at position 48 where a methionine is substituted with a threonine residue. This is a drastic change in physicochemical properties; however, this amino acid residue has not been reported to be of importance for the binding of this subunit to PP1c, therefore we do not expect this change to hamper the inhibitory function of Darpp-32 on PP-1.

### Antibody Validation

**Figure 4:**
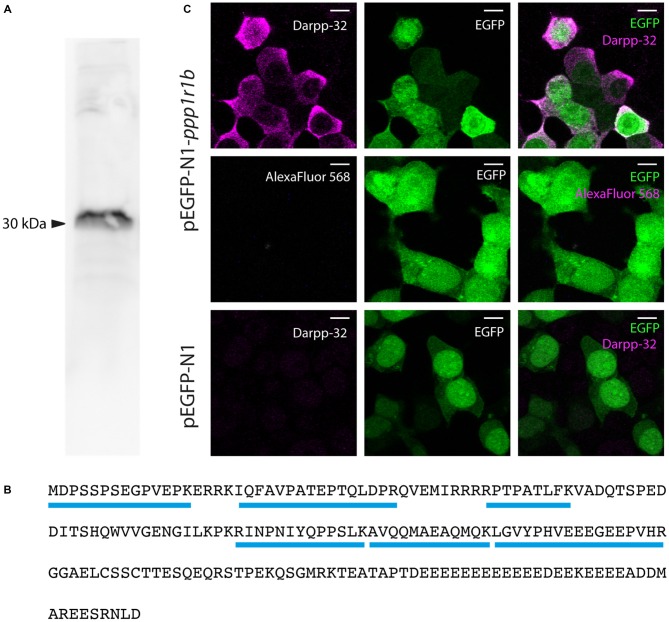
**Validation of the custom-made Darpp-32 antibody. (A)** The anti-Darpp-32 antibody detects one prominent band of approximately 30 kDa on Western Blot of protein lysate of adult zebrafish brain (See also **B**). **(B)** Amino acid sequence of full length Darpp-32. Mass spectrometric analysis of the indicated band in panel 1 detected six peptides unique to full length Darpp-32 (blue bars). **(C)** Upper row: HEK 293T cells transfected with pEGFP-N1-ppp1r1b show that cells showing anti-Darpp-32 immunoreactivity are also enhanced green fluorescent fusion protein (EGFP) positive. Middle row: no staining is seen in the absence of the anti-Darpp-32 antibody. Lower row: HEK 293T cells transfected with pEGFP-N1 without the ppp1r1b insert show no staining. Scale-bar: 10 μm.

We generated a custom antibody against zebrafish Darpp-32 to examine its expression pattern in the larva and adult. This is a polyclonal antibody made by immunizing a rabbit with purified recombinant zebrafish Darpp-32 protein. We tested the specificity of this antibody towards zebrafish Darpp-32 in several ways: first we performed western blot using adult zebrafish brain lysate and the custom Darpp-32 antibody and detected a strong band at around 30 kDa (Figure 4A). To further validate the antibody, we performed immunoprecipitation with anti-Darpp32-antibody-coated beads and adult zebrafish brain lysate, followed by trypsin digestion and mass spectroscopy analysis of the pull down material. Mass spectroscopy identified 6 unique peptides covering 43% of the protein sequence of Darpp-32 (Figure 4B). To demonstrate that the custom Darpp-32 antibody could specifically detect zebrafish Darpp-32 protein, we transfected HEK293T cells with plasmids coding for either a Darpp32-EGFP fusion protein or EGFP alone and then performed fluorescence immunohistochemistry. We find that the Darpp-32 antibody only detects a target in HEK293T cells that were transfected with the pEGFP-N1*-ppp1r1b* plasmid (Figure 4C upper row) but not in HEK293T cells that were transfected with the empty pEGFP-N1 vector (Figure 4C lower row). Staining with anti-rabbit secondary antibody without including the primary antibody against Darpp-32 does not show any signal (Figure 4C middle row).

### Darpp-32 Expression in the Adult Zebrafish Brain

**Figure 5:**
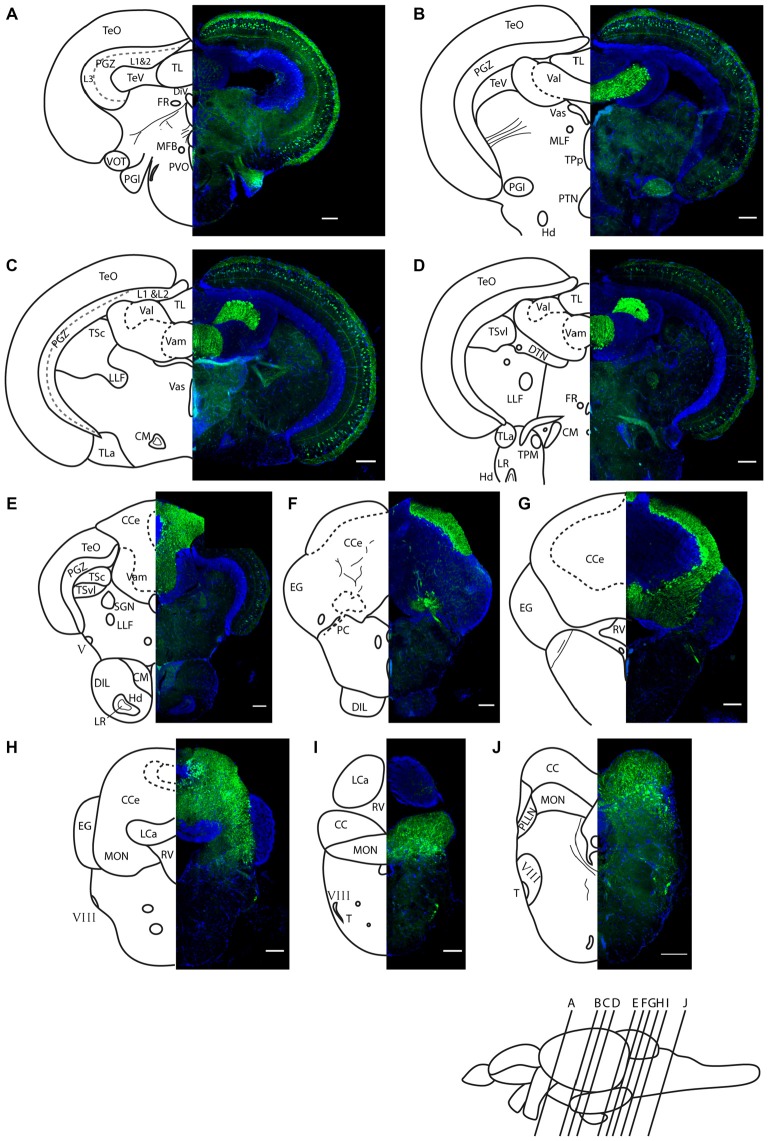
**Darpp-32 immunoreactivity in adult zebrafish brain shown in rostral to caudal coronal sections.** **(A–J)** Averaged *z*-stacks of serial coronal sections taken at the level and angle indicated by lines in the schematic shown in the lower right. Left hand drawings are based on the right hand image. Neuroanatomical structures are labeled according to Wullimann et al. (1996) and personal communication with M. Wullimann. DAPI: blue; anti-Darpp-32: green. Scale bar in all sections: 100 μm. Thin lines in the drawing indicate Darpp-32 immunoreactive fiber tracts. Dashed lines indicate landmarks within a neuroanatomical structure. Abbreviations used in the figure: CC, cerebellar crest; CCe, corpus cerebelli; CM, corpus mamillare; DIL, diffuse nucleus of the inferior lobe; DiV, diencephalic ventricle; DTN, dorsal tegmental nucleus; EG, Eminentia granularis; FR, habenulo-interpenduncular tract; Hd, dorsal zone of periventricular hypothalamus; LCa, caudal lobe of cerebellum; LLF, lateral longitudinal fascicle; LR, lateral recess of diencephalic ventricle; MFB, medial forebrain bundle; MLF, medial longitudinal fascicle; MON, medial octavolateralis nucleus; PC, posterior cerebellar tract; PGl, lateral preglomerular nucleus; PGZ, periventricular gray zone of optic tectum; PLLN, posterior lateral line nerve; PTN, posterior tuberal nucleus; PVO, paraventricular organ; RV, rhombencephalic ventricle; SGN; secondary gustatory nucleus; T, tangential nucleus; TeO, optic tectum; TeV, tectal ventricle; TPM, pretecto-mamillary tract; TPp, periventricular nucleus of posterior tuberculum; TL, longitudinal torus; TLa, lateral torus; TSc, central nucleus of semicircular torus; TSvl, ventrolateral nucleus of semicircular torus; Val, lateral division of valvula cerebelli; Vam, medial division of valvula cerebelli; Vas, Vascular lacuna of area postrema; ventrolateral optic tract; V, trigeminal nerve; VIII, octaval nerve.

**Figure 6:**
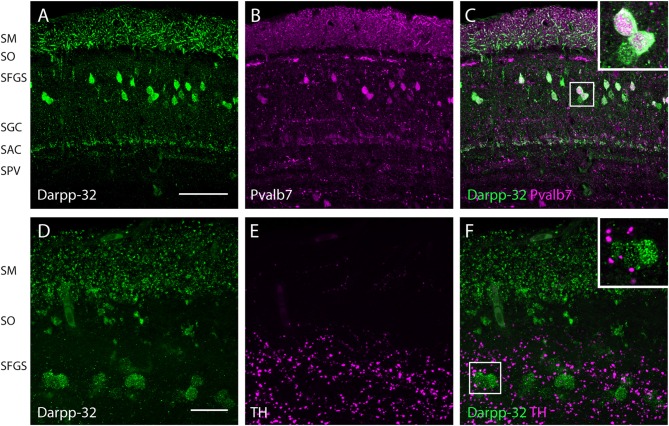
**Expression of Darpp-32, Pvalb7 and tyrosine hydroxylase (TH) in the layers of the adult optic tectum.** Sagittal sections of the adult optic tectum, dorsal to the top. **(A–C)** Colocalization of Darpp-32 and Pvalb7 immunoreactivity in somata in the SFGS and in dendrites in SM. The principal dendrite cannot always be fully seen because it does not necessary lie within the plane of the optical section shown. The layers of the optic tectum are indicated on the left hand side. Scale bar: 20 μm. However, note that the expression of Darpp-32 and Pvalb7 in the synaptic layers ventral to the SM does not overlap. **(D–F)** Intense TH positive fibers are visible in the vicinity of Darpp-32 expressing cell bodies. Scale bar: 50 μm. Abbreviations of the tectal layers from dorsal to ventral: SM, stratum marginale; SO, stratum opticum; SFGS, stratum fibrosum et griseum superficiale; SGC, stratum griseum central; SAC, stratum album central; SPV, stratum periventriculare.

**Figure 7:**
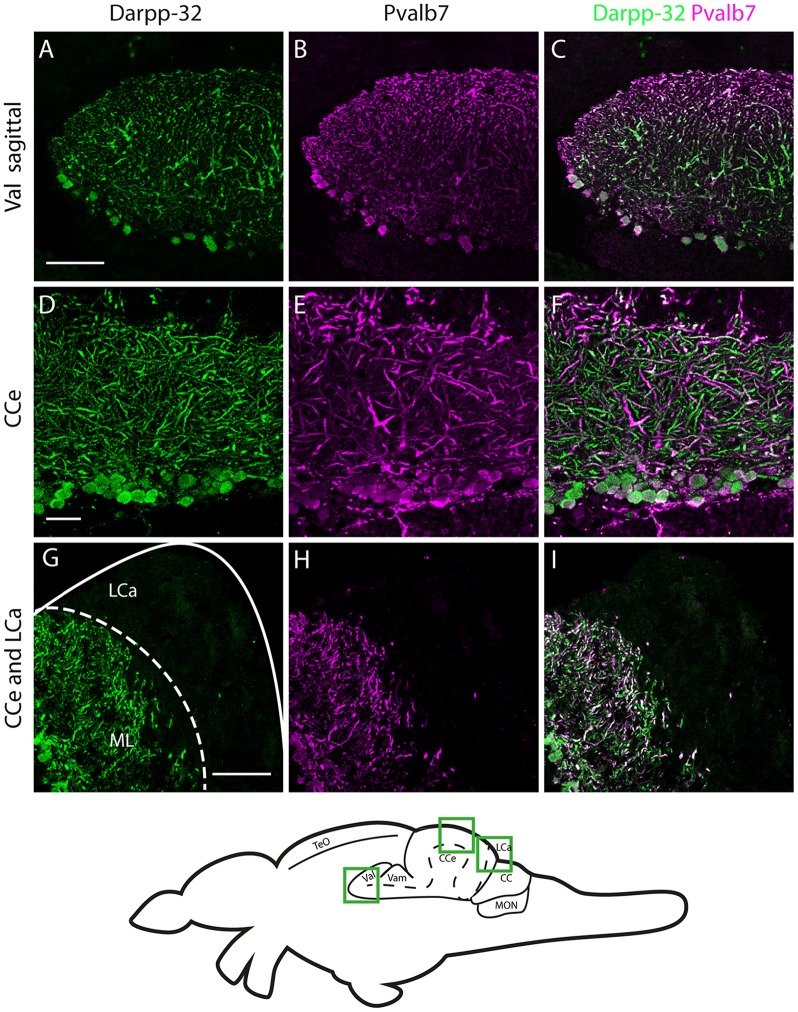
**Darpp-32 and Pvalb7 expression in the cerebellum of the adult zebrafish brain. (A–C)** Sagittal brain slices of the lateral division of the valvula cerebelli (Val). **(D–F)** Purkinje cell layer and molecular layer of the corpus cerebelli (CCe). **(G–I)** The caudal part of the CCe, including the lobus caudalis cerebelli (LCa). The expression of Darpp-32 and Pvalb7 overlaps in all the structures shown, albeit the intensity of expression is heterogenous. Note the restriction of expression of Darpp-32 and Pvalb7 expression to the molecular layer and Purkinje cells in **(G–I)**. The LCa does not show any Darpp-32 staining. Scale bar in **(A–C)** and **(G–I)** is 50 μm. Scale bar in **(D–F)** is 20 μm. The schematic indicates the regions where the images were taken.

**Figure 8:**
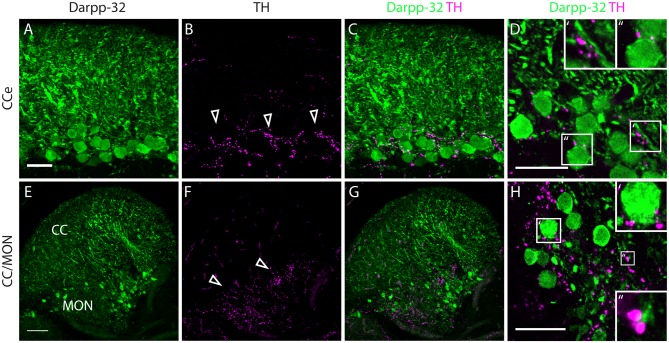
**Darpp-32 and TH expression in the adult zebrafish brain. (A–C)** TH-positive fibers (open arrow heads in **B**) run along the Purkinje cell bodies in the CCe. Collapsed *z*-stacks of confocal images of a coronal section. Scale bar in **(A–C)** 20 μm. **(D)** Higher magnification image showing proximity of TH puncta to Purkinje neuron somata (D′, inset size 11.09× 11.09 μm) and dendrites (D″, inset size 9.98× 9.98 μm). Scale bar in** (D)** 20 μm. **(E–G)** Crest cells in the medial octavolateralis nucleus (MON) express Darpp-32. Their dendrites extend into the cerebellar crest (CC), Darpp-32 positive cell bodies are restricted to the MON. TH positive fibers densely innervate the MON. Mild staining in the TH channel in the CC is unspecific staining of blood vessels. Scale bar in **(E–G)** is 50 μm, sagittal section. **(H)** Higher magnification image showing TH immunoreactive puncta in close proximity to MON crest cells (H′, inset size 9.98× 9.98 μm) and dendrites (H″, inset size 4.28× 4.28 μm), coronal section. Scale bar in **(H)** 20 μm.

We find Darpp-32 to be expressed in the cerebellum and cerebellum-like structures of the adult zebrafish brain (Figures 5–8). To clearly demarcate areas of the zebrafish brain that are positive for Darpp-32, we made thin sections of the brain in coronal and sagittal planes followed by immunohistochemistry with the anti-Darpp-32 antibody. We find strong staining in cell bodies and fiber tracts within the optic tectum (Figures 5A–C), cell bodies and neuropil of the cerebellum in both the corpus cerebelli (CCe) and the Valvula cerebelli (Va; Figures 5B–H) and in the MON (Figures 5H–J). We find no staining in the lobus caudalis (LCa), or the eminentia granularis (EG). No cell bodies were stained in the crista cerebellaris (CC; Figures 5H–J).

In addition to the structures named above, there are a few other areas labeled by the Darpp-32 antibody, albeit with weaker intensity. These structures are the paraventricular organ (PVO), the lateral preglomerular nucleus and some neurons of layer 3 of the periventricular gray zone of the optic tectum (Figure 5A), the lateral longitudinal fascicle (LLF; Figures 5C,D), the corpus mamilare (CM) and the dorsal zone of the periventricular hypothalamus (Hd) close to the lateral recess of the diencephalic ventricle (DiV; Figure 5E) and the VIII nerve and tangential nucleus in the periphery of the medulla oblongata (Figures 5I,J).

### Identification of Cell Types Expressing Darpp-32

In the cerebellum, we found Darpp-32 immunoreactivity in the Purkinje cell layer with somata and spiny dendrites showing very strong staining (Figures 5C–G). From the morphology of these neurons we concluded that Darpp-32 is present in the somata and dendrites of Purkinje neurons. To confirm that this is indeed the case, we performed double labeling of sagittal and coronal sections of the adult zebrafish brain with anti-Darpp-32 and anti-Parvalbumin7 antibodies followed by confocal imaging at high magnification. Parvalbumin 7 has been shown to mark principal neurons in cerebellum and cerebellum-like circuits (Bae et al., 2009; Kaslin et al., 2013).

In the optic tectum, we found colocalization of Darpp-32 and Pvalb7 immunoreactivities, confirming that the Darpp-32 positive neurons are Type I neurons (Figures 6A–C). These neurons receive parallel fiber input from torus longitudinalis (TL) and have dendrites projecting into the stratum marginale (SM) and are called TL-SM-Type I neurons (Bae et al., 2009). The expression of Darpp-32 and Pvalb7 in the synaptic layers of the optic tectum does surprisingly not overlap. There are two distinct lines of Darpp-32 expression visible at the level of the stratum album centrale (SAC; Figures 6A–C). Weak Pvalb7 labeling is visible ventral and dorsal to these two lines but not overlapping (Figures 6A–C and inset). Furthermore, there is strong Pvalb7 staining at the level of type III interneurons (boundary region between SM and stratum opticum (SO)) detectable (Figure 6B). This area is free of Darpp-32 positive staining.

To know whether these Darpp32 immunoreactive cell bodies receive catecholaminergic input, we looked for TH positive fibers in the proximity of Darpp-32 positive staining. We see a high density of TH-positive punctae (Figures 6D–F) in the area of Darpp-32 positive cell bodies. We see punctae instead of fibers, probably because the fibers run perpendicular to the plane of sectioning.

In the cerebellum, Pvalb7 and Darpp-32 are expressed in the same neurons in the Purkinje cell layer of the CCe and the Va, albeit the intensity of immunostaining varies in the dendritic arbors (Figure 7). Taking together the morphological features, the location of the cells and the co-expression of Darpp-32 and Pvalb7 in the same neurons, we conclude that the Darpp-32 expressing neurons in this area are indeed Purkinje neurons. Next, we looked for proximity with TH-immunoreactive fibers and as in the optic tectum, we find TH-positive punctae closely apposed to Purkinje neuron cell bodies and proximal dendrites (Figure 8). We can detect TH-positive fibers in the PCL of the CCe (Figures 8A–D, arrow heads in Figure 8B). The TH positive fibers are confined to the PCL and the proximal granular cell layer (Figure 8D); they do not extend dorsally into the molecular layer.

In the MON, we see Darpp-32 expression in the soma and dendritic arbor of the crest cells. We also see a dense network of TH-positive fibers at the level of Darpp-32 positive cell bodies in the MON but not in the CC (Figures 8E–H).

### Developmental Acquisition of Darpp-32 Immunoreactivity

**Figure 9:**
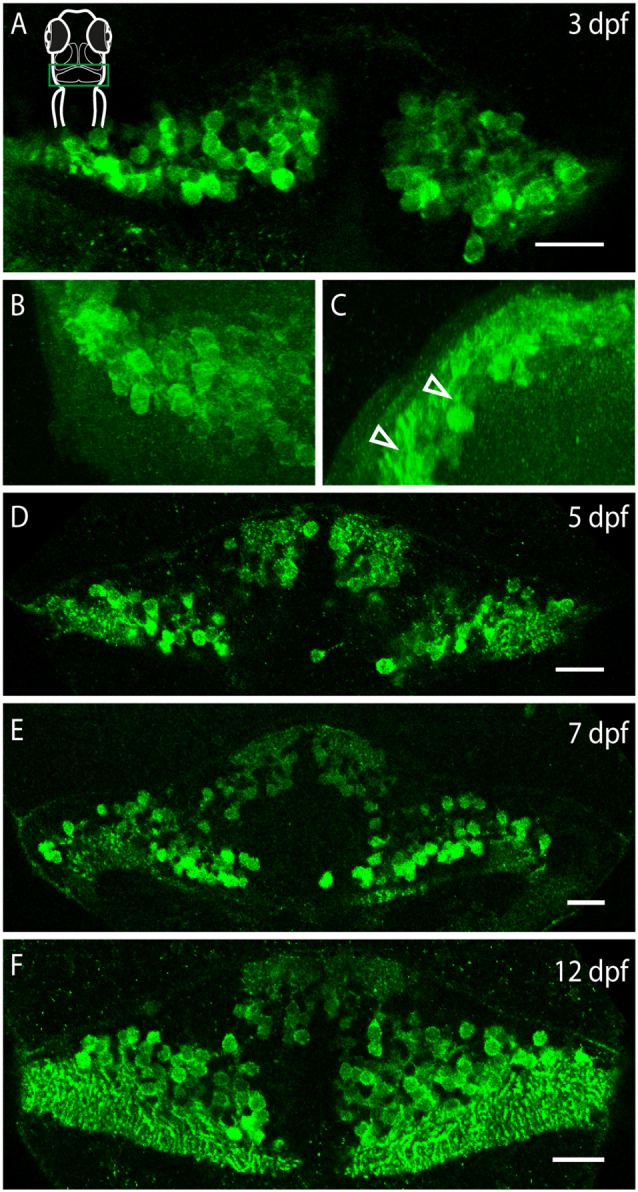
**Darpp-32 expression over the course of development.** Single optical sections of whole mount immunofluorescence stainings **(A,D–F)** and projections of a *z*-stack **(B,C)**. The images are oriented as follows: **(A,B,D–F**) rostral up, **(C)** dorsal up, midline to the right. Schematic in **(A)** is for orientation, the images are showing the cerebellum, indicated by a green box. **(A)** Darpp-32 expression in the cerebellum is visible at 3 days post-fertilization (dpf). At this stage only cell bodies are visible in the dorsal view. **(B)** Dorsal view of a 3D projection of the left hemisphere of a 3 dpf larva. **(C)** Rotation of this 3D-projection around the lateral axis reveals budding dendrites growing towards the dorsal side (white arrowheads). **(D–F)** Darpp-32 continues to be expressed in the cell bodies and dendrites of Purkinje cells throughout the course of development. In **(F)** the outgrowing valvula cerebelli can be seen at rostral end of the cerebellum. Scale bar: 20 μm.

Purkinje neurons are specified beginning at around 3 dpf (Hamling et al., 2015). To investigate when during development Purkinje neurons acquire Darpp-32 immunoreactivity, we performed immunolabeling with the anti-Darpp-32 antibody in larvae from 3 dpf onwards. We found Darpp-32 expression in Purkinje neurons at 3 dpf (Figures 9A–C). A coronal view reveals budding primary dendrites of the developing Purkinje neurons (arrow heads, Figure 9C). The expression of Darpp-32 in the cerebellum remains stable over the course of development (Figure 9). Darpp-32 is expressed in the cytoplasm, nucleus and dendritic arbors of these neurons, with the arbor extent increasing with age. From 7 dpf onwards, the valvula cerebelli starts protruding towards the rostral end. At 12 dpf the zones of dendritic arbors in the rostral and caudal end of the cerebellum are distinct from the cell body layer between those two. This is due to the cerebellar tissue beginning to convolute in the horizontal plane and the images shown are single optical sections across the horizontal plane. The midline remains free of Darpp-32 positive cells throughout. Darpp-32 expression and Pvalb 7 expression colocalize at all developmental stages in the cerebellum (unpublished data).

**Figure 10:**
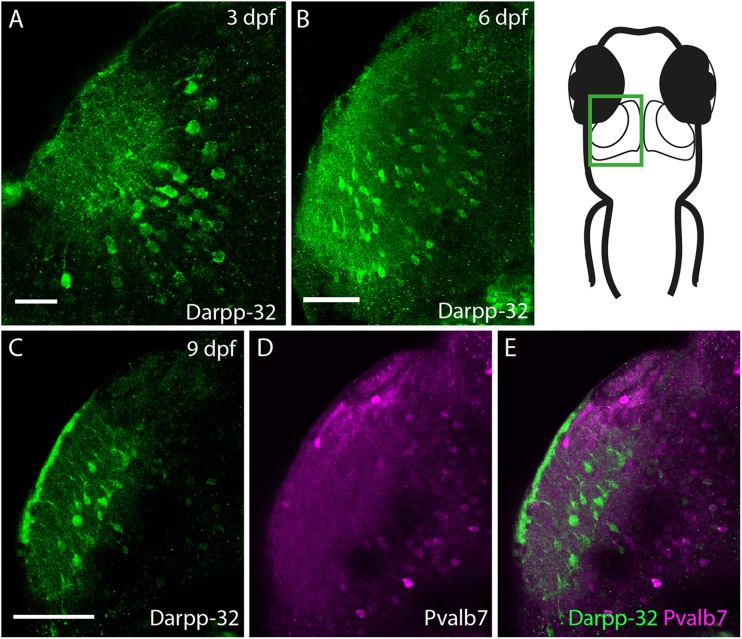
**Darpp-32 expression in the larval optic tectum.** Single optical sections of whole mount immunofluorescence stainings. The images are showing one hemisphere of the optic tectum as indicated in the schematic on the right side. **(A)** Darpp-32 expression in the optic tectum can be first seen at 3 dpf. Darpp-32 is expressed in the neuropil and the cell bodies of projection neurons. **(B)** Darpp-32 expression in optic tectum at 6 dpf. **(C–E)** Note that during the course of development the expression of Pvalb7 **(D)** and Darpp-32 **(C)** does not overlap in the optic tectum **(E)**. In **(C)** intense Darpp-32 expression is visible in the superficial most layer, presumably the SM, while Pvalb 7 expression is detectable more closer to the midline, presumably in neurons of the stratum periventriculare (SPV). Darpp-32 positive cell bodies located are more lateral compared to Pvalb 7 positive ones. Scale bar in **(A)** 20 μm; in **(B–E)** 50 μm.

Darpp-32 is also expressed in the larval optic tectum, starting from 3 dpf (Figure 10A). Darpp-32 is visible in the neuropil and in cell bodies of the optic tectum. The dendritic extensions project towards the dorsal lateral side of the optic tectum. The Darpp-32 positive cell bodies are at this stage only present in the neuropil region (Figures 10A,B, region bounded by inner curve in the schematic). Interestingly the expression of Darpp-32 and Pvalb7 during development does not overlap. At 9 dpf intense Darpp-32 expression is present more laterally compared to Pvalb7 (Figures 10C–E). This could be the developing SM. Pvalb7 positive cells are located more towards the midline in relation to the Darpp-32 positive cells.

## Discussion

Here we show that the PP-1 inhibitor Darpp-32 coded for by the *ppp1r1b* gene is expressed preferentially in the principal neurons of the cerebellum and cerebellum-like circuits. Darpp-32 immunoreactivity is intense in Purkinje neurons, crest cells of the MON and TL-SM-Type I neurons of the optic tectum. We identified these neurons by their expression of Pvalb7. We also found that these neurons are likely to receive direct catecholaminergic innervation. These data show that Darpp-32 is a common signal integrator in principal neurons of the cerebellum and cerebellum-like circuits in zebrafish where it might mediate catecholaminergic neuromodulation. Darpp-32 may also be involved in mediating signaling via other GPCRs in these circuits.

### Conserved Features of Darpp-32 Sequence and Function

Genome and transcriptome sequence homology shows *ppp1r1b* to be present in all jawed vertebrates including cartilaginous fishes but absent in invertebrates and agnathans (Yger and Girault, 2011). Thus Darpp-32 could have evolved in early gnathostomes as a ligand-regulated signal-integrating molecule in the CNS. Consistent with this, we provide the first experimental evidence for the existence and CNS-specific localization of Darpp-32 in zebrafish, which are bony fishes and diverged from mammals roughly 450 million years ago. We report that the zebrafish full-length Darpp-32 contains the motif for binding the catalytic domain of PP-1 and the phosphorylation site critical for PP-1 inhibition (Figure 3). Thus, most likely, the full length Darpp-32 protein in zebrafish is also an inhibitor of PP-1. We have shown that all neurons that are positive for Darpp-32 are also likely to receive direct catecholaminergic innervation. Taken together, these results point to a conserved function of Darpp-32 in mediating PP-1 inhibition after activation of aminergic GPCR receptors. This also indicates the early evolutionary origins of Darpp-32 function in PP-1 inhibition.

However, serine residues that are important for regulating the inhibitory effect of Darpp-32 towards PP-1 are absent or present in non-conserved regions in zebrafish (Figure 3). These include Serine 102, which is phosphorylated by CK2 and Serine 137 (129 in zebrafish), which is phosphorylated by CK1. Phosphorylation at both these residues acts to potentiate Darpp-32’s inhibitory action on PP-1 (Greengard et al., 1999). It is possible that the activity of Darpp-32 in zebrafish is regulated through alternate mechanisms. While the N-terminus half is highly conserved, there is considerable sequence variation in the C-terminus half of the zebrafish protein compared to mammals (Figure 3). The functional implication of this variation needs to be deciphered.

### Comparative Analysis of Darpp-32 Expression in Cerebellum and Cerebellum-Like Structures Among Vertebrates

Darpp-32 was first isolated from rodent striatal extracts and was shown to be enriched in dopaminoceptive regions of the brain (Walaas et al., 1983a,b). Using antibodies specific to Darpp-32, the protein was detected in the neostriatum of representative mammalian species (Ouimet et al., 1984, 1992). Darpp-32-like immunoreactivity was also detected in the CNS of amniote vertebrates such as birds (Durstewitz et al., 1998; Reiner et al., 1998; Absil et al., 2001) and reptiles (Smeets et al., 2001, 2003) as well as anurans (Lopez et al., 2010). Previously, Darpp-32 was reported to be absent in teleosts based on immunohistochemistry using antibodies raised against the bovine protein (Hemmings and Greengard, 1986). Based on this, it was suggested that Darpp-32 evolved later during vertebrate evolution (Hemmings et al., 1987). Our results support the view that Darpp-32 evolved as an inhibitor of PP-1 in early jawed vertebrates. Consistent with this view, the expression pattern of Darpp-32 is conserved in many brain regions across vertebrates. For example, Darpp-32 expression is observed in the tectum of bony fishes (zebrafish, this study), amphibians (Rana and Xenopus, Lopez et al., 2010), reptiles (turtles, Smeets et al., 2003; lizards, Smeets et al., 2001) and birds (quail, Absil et al., 2001; chick, Araki et al., 2000; and Metzger et al., 2006). Similarly, expression in Purkinje neurons is present in fish (this study), amphibians (Rana, Lopez et al., 2010) and mammals (rats, Ouimet et al., 1984; monkeys, Ouimet et al., 1992). These observations lend support to the idea that Darpp-32 is a conserved molecular signature of cerebellum and cerebellum-like circuits across jawed vertebrates. Nevertheless, Darpp-32 expression is absent in the cerebellum of birds and reptiles and absent in the sole cerebellum-like circuit of mammals, the dorsal cochlear nucleus (Bogush et al., 2005; Bell et al., 2008). Based on these comparisons and our data, we propose that Darpp-32 could have been a marker for cerebellum and cerebellum-like circuits in the early, jawed vertebrates which was then selectively lost in some of these circuits in present day vertebrates.

### Darpp-32 and the Striatum in Zebrafish

Darpp-32 is a marker for MSNs of the striatum. Striatal MSNs use substance-P, enkephalin, dynorphin and GABA as neurotransmitters and receive dopaminergic input via D1/D2 receptors. Expression of these molecules has been used to demarcate areas corresponding to striatum in diverse species of teleosts including zebrafish (Wullimann and Mueller, 2004; Mueller et al., 2006). Based on this, it has been proposed that the zebrafish striatum lies within the dorsal and central nuclei of ventral telencephalon (Wullimann and Mueller, 2004; Laure and Vernier, 2010). Expression patterns of striatal-specific transcription factors in these areas appears to be consistent with this view (Ganz et al., 2012). Though we observed catecholaminergic innervation in this region, we failed to detect Darpp-32 positive neurons in these nuclei (data not shown). It is possible that dopaminergic neuromodulation of striatal circuits in zebrafish is mediated via other signaling pathways. In fact, Darpp-32 positive neurons were absent in several other nuclei known to receive dopaminergic inputs such as the reticulospinal network in the rhombencephalon. Activation of D1 receptors recruits signaling via PKA, phospho lipase C (PLC), and extracellular signal regulated kinases (ERK) pathways. Similarly, activation of D2 receptors is known to inhibit PKA, activate PLC and inhibit Akt pathways (Beaulieu and Gainetdinov, 2011). In the absence of Darpp-32, these additional pathways can mediate signaling via dopaminergic and adrenergic receptors.

### Darpp-32 mRNA and Protein Expression during Development

We isolated three different transcripts of *ppp1r1b* with two TSSs (Figure 1). Of these, *ppp1r1b-001* codes for the full length Darpp-32 protein while *ppp1r1b-002* and *ppp1r1b-003* code for shorter amino acid chains. While *ppp1r1b-001* and *ppp1r1b-003* are maternally deposited and continue to be expressed throughout all stages of development, the onset of expression of *ppp1r1b-002* is late (Figure 1B). During initial stages of development, the full-length transcript is expressed ubiquitously, with the region of expression getting restricted at later stages of development (Figure 2). However, the protein is first detectable in the CNS only at 3 dpf. This is an unusual delay between detection of transcript and detection of protein, assuming that all three isoforms of Darpp-32 can potentially be expressed in the CNS. At this point we are not able to distinguish between the three isoforms at the mRNA or protein level—the unique regions in the mRNA are too short for designing probes and the antibody we used detects all three isoforms (data not shown).

The onset of Darpp-32 expression in Purkinje neurons of zebrafish coincides with Pvalb7 expression (unpublished data) and the reported onset of Zebrin II expression (Bae et al., 2009). While Zebrin II is expressed only postnatally in rodents and is therefore considered to be a late-onset marker for Purkinje neurons (Larouche and Hawkes, 2006), it is an early-onset marker in zebrafish as its expression coincides with the beginning of differentiation of Purkinje neurons (Bae et al., 2009; Hashimoto and Hibi, 2012). Like Pvalb7 and Zebrin II, Darpp-32 is expressed from the onset of Purkinje neuron differentiation with a seemingly unchanging expression profile from developing to mature Purkinje neurons.

### Darpp-32 and Purkinje Neuron Activity

Our recent work shows that Purkinje neurons *in vivo* are bistable and that climbing inputs mediated by AMPA type glutamate receptors (AMPAR) on Purkinje neurons triggers bursting in these neurons (Sengupta and Thirumalai, 2015). AMPARs are known targets for modulation by the Darpp-32-PP-1 pathway. When D1 receptors are activated, increased PKA activity causes phosphorylation of both AMPARs as well as Darpp-32. In turn, Darpp-32 prevents dephosphorylation of AMPARs by inhibiting PP-1 (Greengard et al., 1999). As a result, AMPARs are maintained in a phosphorylated state leading to enhanced channel currents. Taken together, the above results implicate Darpp-32 in regulating bistability in Purkinje neurons by modulating AMPAR-mediated synaptic transmission. This will have to be tested in future studies.

### Functional Relevance of Darpp-32 in Cerebellum-Like Circuits

**Figure 11:**
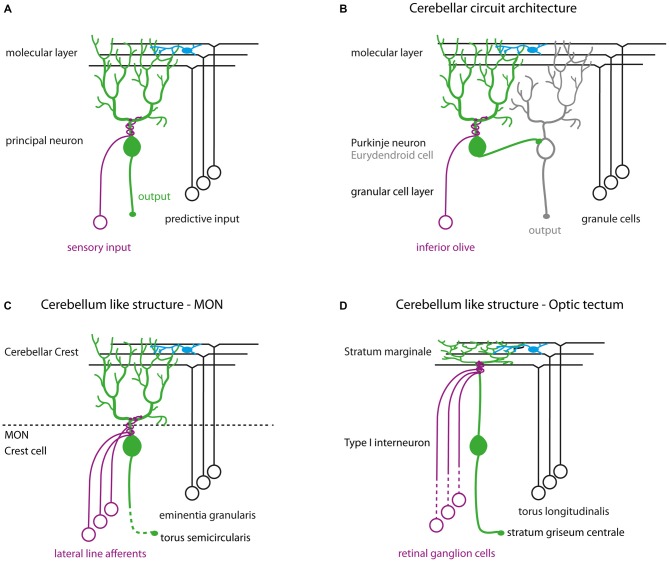
**(A)** Schematic of cerebellum-like circuits. Green cells indicate principal neurons in cerebellum-like circuits that express Darpp-32. Blue cells in all schematics represent stellate cells. **(B)** Cerebellar circuit architecture in teleosts. **(C)** Cerebellum-like circuitry in the MON. **(D)** Cerebellum-like circuitry in the optic tectum.

We show here that Darpp-32 is preferentially expressed in principal neurons of cerebellum-like circuits. Cerebellum-like circuits share a basic wiring diagram consisting of principal neurons receiving parallel fiber inputs on distal dendrites in the molecular layer and peripheral input on proximal dendrites (Figure 11A; Bell, 2002; Bell et al., 2008). These principal neurons can be excitatory such as the MON crest cells and the optic tectal TL-SM-Type I neurons or inhibitory like Purkinje neurons in the Va and CCe (Figures 11B–D). Each of these circuits receives distinct peripheral inputs on their basilar dendrites: the MON receives signals from the lateral line system, the TL-SM-Type I neurons receive retinal inputs while the Purkinje neurons receive inputs from the inferior olive. Similarly, the origin of parallel fiber inputs onto these principal neurons is also distinct (Figure 11). Thus, the function performed by these circuits must also be different from each other: the cerebellum is implicated in motor control, the MON in mechanosensation and the tectum in visual processing. However, the shared circuit template in these divergent circuits suggests shared computational principles operating in these circuits. In this respect, we find it significant that a signal-integrating molecule such as Darpp-32 is preferentially expressed within principal neurons of these homologous circuits. Cerebellum-like circuits have a high degree of input convergence and the presence of a signal-integrating molecule like Darpp-32 may be vital for processing the barrage of neurotransmitter and neuromodulator inputs.

## Author Contributions

LR: designed experiments, performed experiments, analyzed data, created images, wrote the manuscript. VT: designed experiments, analyzed data, wrote and edited the manuscript.

## Conflict of Interest Statement

The authors declare that the research was conducted in the absence of any commercial or financial relationships that could be construed as a potential conflict of interest.

## Footnotes

^1^www.ensembl.org

^2^www.benchling.com

^3^http://www.ebi.ac.uk/

^4^www.fiji.sc

^5^www.ensembl.org

^6^www.ncbi.nlm.nih.gov

^7^http://www.uniprot.org/
